# Selective Wine Aroma Enhancement through Enzyme Hydrolysis of Glycosidic Precursors

**DOI:** 10.3390/molecules29010016

**Published:** 2023-12-19

**Authors:** José Manuel Rodríguez-Nogales, Encarnación Fernández-Fernández, Violeta Ruipérez, Josefina Vila-Crespo

**Affiliations:** 1 Food Technology Department, Higher Technical School of Agrarian Engineering of Palencia, University of Valladolid, Av. Madrid 50, 34004 Palencia, Spain; encarnacion.fernandez@uva.es; 2 Microbiology Department, Higher Technical School of Agrarian Engineering of Palencia, University of Valladolid, Av. Madrid 50, 34004 Palencia, Spain; violeta.ruiperez@uva.es (V.R.); josefinamaria.vila@uva.es (J.V.-C.)

**Keywords:** aroma, glycosidases, white wine, varietal aroma, terpenes

## Abstract

Selective enhancement of wine aroma was achieved using a broad spectrum of exogenous glycosidases. Eight different enzyme preparations were added to Verdejo wine, resulting in an increase in the levels of varietal volatile compounds compared to the control wine after 15 days of treatment. The enzyme preparations studied were robust under winemaking conditions (sulfur dioxide, reducing sugars, and alcohol content), and no inhibition of β-glucosidase activity was observed. Significant differences were detected in four individual terpenes (α-terpineol, terpinen-4-ol, α-pinene, and citronellal) and benzyl alcohol in all the treated wines compared to the control wine, contributing to the final wine to varying degrees. In addition, a significant increase in the other aromatic compounds was observed, which showed different patterns depending on the enzyme preparation that was tested. The principal component analysis of the data revealed the possibility of modulating the different aromatic profiles of the final wines depending on the enzyme preparation used. Taking these results into account, enhancement of the floral, balsamic, and/or fruity notes of wines is possible by using a suitable commercial enzyme preparation.

## 1. Introduction

Wine aroma comprises a wide variety of compounds with different aromatic properties, which arise from the interaction between grape components and those produced during processing, fermentation, and aging [1]. Some of these compounds exist in free volatile form, while others accumulate in grape berries as odorless, non-volatile, and flavorless precursors, mainly glycosides [1,2]. Glycosides are, in most cases, more abundant than unglycosilated forms of individual terpenes [3,4]. These glycoconjugates comprise an aroma compound (aglycone) bound to a sugar moiety. The aglycone part of glycosides includes monoterpenes, C13-norisoprenoides, benzene derivatives, and aliphatic alcohols. The sugar moiety is represented by glucose or disaccharides [2]. To enrich wine aroma through the release of free aromatic compounds from glycoside precursors, acidic or enzyme hydrolysis is required. Acidic hydrolysis simulates the reactions taking place during the aging of wines, producing different patterns of volatile monoterpenes depending on pH conditions. Enzyme hydrolysis appears to be a more natural and more efficient method to enhance terpenes in the wine. Glycosidases with oenological implications have been described in grapes, yeast, bacteria, and fungi, suggesting that this is a promising approach [5,6]. Various enzymes can act in sequence in the case of diglycosides. The reaction can undergo a two-step hydrolysis through the action of an appropriate glycosidase (arabinofuranosidase, rhamnopyranosidase or apiofuranosidase) to release the terminal sugar (arabinose, rhamnose, or apiose, respectively) followed by β-glucosidase action to release the bound volatile compound. For monoglucosidic precursors, only the action of β-glucosidase is needed [5,7,8].

The potential for β-glucosidase activity to be used in the food industry and, specifically, in wine aroma enhancement has been studied [9]. Although β-glucosidase is present in grapes, its activity is insufficient due to its low stability under juice-processing and winemaking conditions [3,10]. Therefore, microorganisms have been considered the main source of this enzyme [9]. Activity varies significantly depending on the yeast species, and some strains are not able to produce this enzyme at all. Furthermore, enzymes may be produced in different localizations inside the cell, so the intracellular β-glucosidase activity is of little significance for industrial applications. In this sense, research needs to focus on yeasts producing extracellular β-glucosidase [11]. 

*Saccharomyces cerevisiae* displays low levels of β-glycosidase activity under fermentation conditions and has minimal ability to produce extracellular β-glucosidase, resulting in limited or even negligible β-glucosidase yield and activity. In general, high β-glucosidase activity is uncommon in *S. cerevisiae* strains [11]. However, the presence of exo-1,3-β-glucanase activity in *S. cerevisiae* has been described. The use of a recombinant *S. cerevisiae* strain overexpressing exo-1,3-β-glucanase activity encoded by the EXG1 gene achieved higher levels of free volatiles in wine. The inoculation of this strain resulted in the increment of some alcohols and terpenes, although no increase in one of the most abundant terpenes in grapes, linalool, was detected [12]. However, the approach of increasing wine aroma using genetic engineering techniques is subject to social controversy and legal restrictions. 

To overcome the limited effect of glycosidases from grape and *S. cerevisiae* yeasts, the addition of exogenous glycosidases has been pointed out to enhance wine aroma [9,11]. The use of exogenous glycosidases in winemaking has been mostly focused on grape varieties rich in glycosides of monoterpenes in order to achieve a quick release of aromatic volatile compounds beyond threshold levels [2]. Various studies have reported the ability of specific enzymes derived from microorganisms to produce higher levels of aromatic compounds. Interestingly, several commercial enzyme preparations designed specifically to improve wine aroma are available for winemaking.

Several exogenous enzymes with a fungal origin have been developed to release glycoside precursors in wines. Exoglycosidases and β-glucosidase from *Aspergillus niger* have been shown to be remarkably stable in wine pHs, in contrast to those from *S. cerevisiae* [1]. β-glycosidase from a strain of *Aureobasidium pullulans* isolated from grape ecosystems showed a wide range of pH stability, tolerance to low temperatures and ethanol, and the ability to efficiently release free terpenols [13]. These authors also identified a basidiomycetous yeast in oenological ecosystems, *Sporidiobolus pararoseus*, to be used as a source of β-glucosidase. The authors reported that the enzyme remained stable under winemaking conditions and the enzymatic treatment led to an increase in the amount of unbound terpenes [14]. A different approach was developed by Zhu et al. [15] to investigate the release of glycosidically bound aroma compounds by adding exogenous β-glucosidase. Crude extracts obtained from a high-producing β-glucosidase-fused protoplast of *A. oryzae* and *A. niger* were successfully used to enhance aromatic compounds through the hydrolysis of glycosidic precursors [15]. The isolation of β-glucosidases from non-*Saccharomyces* yeasts has also been reported. On the one hand, isolated enzymes from *Meyerozyma guilliermondii* NM218 and *Hanseniaspora uvarum* BF345 were used in young Chardonnay wines, achieving high specificity and maintaining activity during wine aging [16]. On the other hand, the addition of purified β-glucosidases from *Issatchenkia terricola*, *Pichia kudriavzevii*, and *Metschnikowia pulcherrima* to the must produced high levels of terpenes, improving the flavor complexity and quality of the wine [17]. Some authors have suggested that the use of indigenous strains as a source of β-glucosidases has the advantage of improving the aromatic profile while maintaining the distinctive characteristics of the wine [10]. Moreover, the performance of β-glucosidases is dependent on the enzyme’s resistance to winemaking conditions. Differences in their ability to hydrolyze aromatic precursors have also been described [10]. 

Commercial enzyme preparations are usually mixtures of several enzymes, and the side effects of these enzymes have been reported to affect the wine in either a positive or negative way [10,18]. Therefore, screening is necessary to select the most suitable enzyme to improve the aromatic profile of a specific type of wine. Commercial products including β-glucosidase activity with other enzymes are available for winemaking. However, information regarding which enzymes are more suitable for a specific need may not always be available or well organized [9]. As these preparations differ significantly in glycosidase activities, research in this area is necessary for characterizing their impact on wine aroma profiles. The use of commercial glycosidase preparations in winemaking has been applied to increase the wine flavor in some grape varieties, obtaining different sensorial attributes from those of the control wines [2,19]. 

The aim of this work was to evaluate the effectiveness of glycosidases in the selective enhancement of the floral, balsamic, and/or fruity aroma of wine. For that purpose, a range of enzyme preparations have been tested, and the varietal volatile compounds of the treated wines have been analyzed. These results are of considerable practical interest in order to produce wines with controlled aromas by means of the selective hydrolysis of glycosidic aroma precursors. 

## 2. Results and Discussion

### 2.1. Effect of Reducing Sugars, Alcoholic Content, and Sulfur Dioxide on β-Glucosidase Activity

Among the enzymes involved in the release of aromatic compounds, β-glucosidases (β-d-glucopyranoside glucohydrolases, E.C. 3.2.1.21) play a crucial role in the hydrolysis of non-volatile compounds in must and wine [10,20,21]. It has been previously reported that wine conditions such as high levels of glucose and ethanol content, pH, and sulfur dioxide may affect β-glucosidase activity [10,11]. Therefore, the study of the activity at wine pH, high sugar, and ethanol concentrations and the presence of sulfites is required for their use in the wine industry [9].

The potential of enzymes isolated from yeasts and fungi tolerant to high glucose and ethanol concentrations has been previously described [13,14,22]. To study the interplay of reducing sugars, alcoholic degree, and free sulfur dioxide, β-glucosidase activity from the commercial enzyme extracts was assayed in samples of wine (pH 3.6) with three different concentrations of free sulfur dioxide (12, 36, and 60 mg/L), reducing sugars (1.8, 5.9, and 10.0 g/L glucose), and alcoholic degree (10.0, 12.5, and 15.0% *v*/*v*). The levels selected for each parameter are the most common during the winemaking of white wines. For this study, a Box–Behnken design was applied [23]. The advantage of the Box–Behnken design is the reduced number of experiments required. Moreover, it is more efficient and easier to arrange and interpret in comparison to other designs [24]. 

The results of β-glucosidase activity found in the enzyme extracts (Table 1) showed significant differences among the commercial enzyme preparations, ranging from averages of 69.153 U to 4.093 U, except for E7 and E8, which displayed similar activity. The differences found in β-glucosidase activity can be attributed mainly to the enzyme composition of the commercial preparations, which are isolated from different sources and comprise a mixture of enzymes [1,9,21]. It has been reported that the presence of glucose does not affect β-glucosidases isolated from fungi and yeasts in the same way, showing different inhibition patterns [14]. Interestingly, distinct β-glucosidases isolated from the same source have shown unequal tolerance to glucose levels [5]. 

Generally, the activity of this enzyme is reduced because of the high levels of glucose in must [11]. The incidence of the reducing effect of high glucose concentrations on β-glucosidase activity depends on the enzyme characteristics, affecting the structure of the protein in a reversible manner [10]. In this sense, β-glucosidases have shown the ability to catalyze reverse hydrolysis and transglycosylation reactions, synthesizing oligosaccharides and glycosides [25,26]. Regarding different commercial preparations, the addition time is recommended by the manufacturer depending on this property. It has been found that some of them need to be added at the end of fermentation to prevent sugar inhibition [9]. In addition, as alcoholic fermentation takes place, the increase in ethanol content can also affect the activity of β-glucosidases [11]. In the same way, during the aging of wines, the high ethanol content can exert an inhibiting effect on β-glucosidase activity [16]. Regarding sulfur dioxide, research on the influence of β-glucosidase activity is very limited, and further studies of its combined effect with other oenological conditions are required [11].

**Figure 1:**
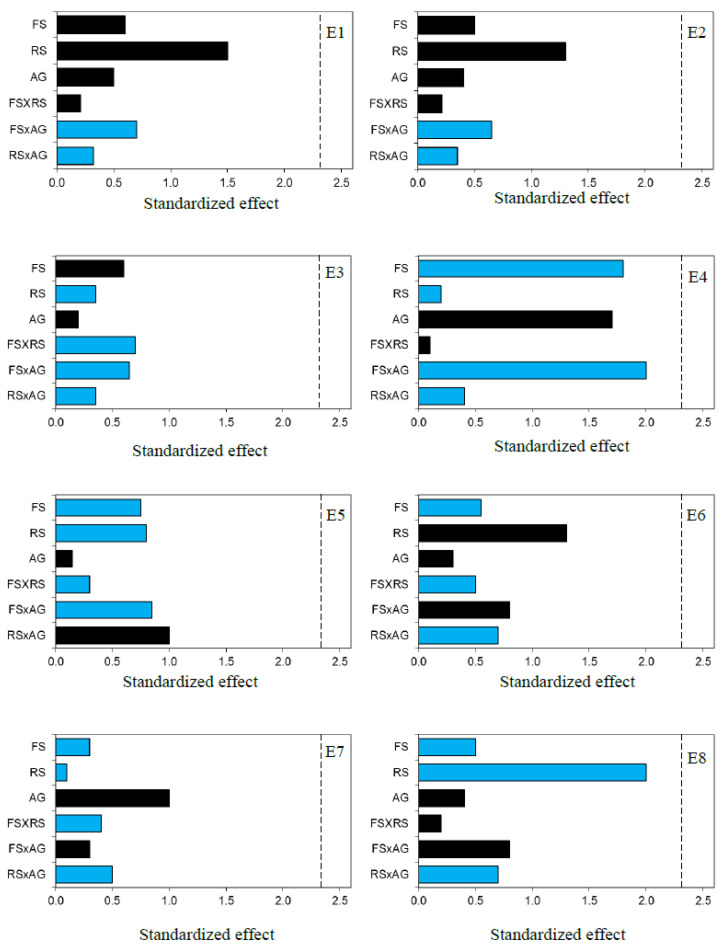
Pareto chart of Box–Behnken design for β-glucosidase activity of the commercial enzyme preparations (E1–E8) (FS: free sulfur dioxide; RS: reducing sugars; AG: alcoholic grade). Black and blue bars represent positive and negative standardized effect values, respectively. Factors that extend beyond the vertical dashed line are significant at *p* < 0.05.

The plot of the standardized effect of each of the parameters investigated on the β-glucosidase activity of the enzyme extracts was performed (Figure 1). In this treatment, a parameter is considered to have a significant influence if its standardized effect is over 2.3. The dataset analysis revealed that the levels of reducing sugars, alcoholic grade, and sulfur dioxide assessed did not significantly affect or modify the β-glucosidase activity of the enzyme preparations. In this respect, the low standard deviation of the β-glucosidase activity of the enzyme preparations (Table 1), obtained from the enzyme activities measured under the different oenological conditions (trials 1–15), also indicates the high efficacy and stability of each of the enzyme preparations.

In contrast to our data, other authors have shown that β-glucosidase activity in commercial enzyme preparations was reduced at high ethanol concentrations or in the presence of glucose, with the extent of inhibition depending on the source of the enzyme [18,27]. A recent study also described the characterization of a cold-active β-glucosidase, which showed a marked inhibition by glucose levels. In contrast, high activity was found in the presence of fructose (10–200 g/L), ethanol (10–25% *v*/*v*), and sulfur dioxide (30 mg/L), which did not affect enzyme activity [21]. 

After our results, it is possible to affirm that the commercial enzyme preparations tested in this study are robust from an oenological point of view and can be successfully used under the tested winemaking conditions.

### 2.2. Effect of Glycosidase Enzyme Treatment on Wine Composition and Aroma

The general composition of the control and enzyme-treated wines is shown in Table 2. Enzyme treatments had no significant effect on the general composition of the wines, except for reducing sugars and free sulfur dioxide (*p* < 0.05). No significant differences were found in pH, total and volatile acidity, or total sulfur dioxide. In agreement with our data, a previous study focused on the effect of five commercial preparations on white wine composition showed that enzymes do not have a significant influence on the basic physicochemical parameters [28]. However, regarding the reducing sugar concentration, an increase in this parameter was observed in the wines treated with enzymes (2.9 g/L glucose, average of enzyme-treated wines) in relation to the control wine (1.3 g/L glucose). This effect has also been reported by Martino et al., who observed an increment in glucose release in white wine treated with an industrial extract from *A. niger* as a result of residual glucosidase activity [29]. This increment in reducing sugars can be explained by the ability of this enzyme to release oligosaccharides upon the hydrolysis of glycosidic bonds [11,25]. In this sense, β-glucosidases have also been used in the sugar production industry [9]. The enzyme treatment also resulted in a decrease in free sulfur dioxide from 40 mg/L (control wine) to 31 mg/L (average of the enzyme-treated wines), which may be due to a possible combination of sulfur dioxide with the reducing sugars [30,31] coming from enzyme hydrolyses. 

It is widely accepted that wine aroma and flavor properties are the consequence of many volatile compounds and rarely depend on a single dominant compound. Volatile compounds come from several sources, such as grape berries, yeast and bacteria metabolism, oak wood, chemical reactions or enzymes, and chemical hydrolysis of non-volatile precursors [32]. In this study, we have focused our attention on the enzyme-derived compounds released through the treatment of musts with different commercial preparations to enhance and modulate fruity, floral, and balsamic flavors in wines.

The volatile organic compounds (VOCs) detected in the wines were grouped into three categories according to structural similarities: terpenes, C13-norisoprenoides, and alcohols. The use of commercial enzyme preparations resulted in an enhancement of wine’s aromatic properties (Table 3). Significant differences in four individual terpenes were detected in all the treated wines compared to the control wine: α-terpineol, terpinen-4-ol, α-pinene, and citronellal. The presence of these compounds gives the final wine floral, fruity, citrus, and woody notes [33,34,35] to varying degrees in all the treated samples compared to the control. In addition, eucaplytol increased moderately after treatment with E1, E3, and E8, as did eugenol in all the samples except for E4 and E5. The presence of these compounds may confer balsamic, clove, and herbaceous notes to the final wines [33,34]. The high levels of methyl eugenol in E7 and E8, citronellol in E3, E4, and E8, linalool in E1 and E4, and limonene in E1, E2, and E3 can be associated with floral, citrus, and fruity aromas. Methyl eugenol is regarded as a semi-volatile compound that is naturally present in a variety of diverse food sources and exhibits floral characteristics [36,37], while citronellol, linalool, and limonene are associated with citrus, clove, fruity, green, and floral notes [16,33]. 

Other authors have previously studied the effect of commercial enzyme preparations on the aroma profile of wine. The influence of five commercial enzyme treatments with pectinolytic and glycosidase activities on volatile compound families, other than terpenes and C13-norisopronodeis, revealed a variable evolution of these compounds during alcoholic fermentation depending on the type of enzymes added [28]. These authors reported that enzyme treatments increased the volatile compounds compared to the control. Significant differences in the quality of the final wines were also found, depending on the type of enzyme used [28]. Enzyme-treated wines with the commercial preparation AR2000, which possesses relevant glucosidases, enhanced the attributes of honey, lime, and smoky and were preferred to the control wines [2]. Increments of α-terpineol, linalool, nerol, and geraniol upon hydrolysis of the commercial enzyme preparation AR2000 have also been reported from Muscat glycoside extract [12]. A recent study compared the effect of three isolated β-glucosidases and the commercial preparation AR2000 on the aromatic profiles of Cabernet Gernischt. In this study, the authors reported that differences in the enhancements of volatile compounds, including C_13_-norsisoprenoids, terpenes, and C_6_ compounds, depended on the enzyme used. Interestingly, the analysis revealed that most of the compounds detected in the treated wines were also present in the untreated sample [10]. This result can explain the unequal volatile compounds found in different studies, indicating the great influence of grape variety and its potential to provide aromatic precursors. Differences in the profiles of glycosidic precursors among grape varieties can also vary depending on vine cultivation conditions, weather, or winemaking practices [4,38]. Furthermore, there are variations between different parts of the grape bunch or even in the berry itself [4]. 

A significant increase in benzyl alcohol was found in all the treated wines compared to the control. The various enzyme preparations contributed varying amounts of this compound to the final wine. Benzyl alcohol has been associated with toasted, almond, and fruity aromas when its concentration is above 200 mg/L [16,34]. Therefore, although the increase in this alcohol in wines is significant compared to the control, the concentrations found after treatment are far from being above the established odor thresholds. It is considered that the characteristic aroma of the product is provided above the threshold levels. Below these levels, the compound may contribute to the overall aroma of the wine by interacting with other molecules [33]. 

Changes in the aromatic composition of white wine after must treatment due to the presence of individual alcohols as a side effect of enzymes present in blended commercial enzyme preparations have been described previously [18]. Other authors also reported the effect of the commercial enzyme preparation AR2000 on the concentration of the alcohols in Muscat glycoside extract, leading to an increase in benzyl alcohol and 2-phenylethanol [12]. 

**Figure 2:**
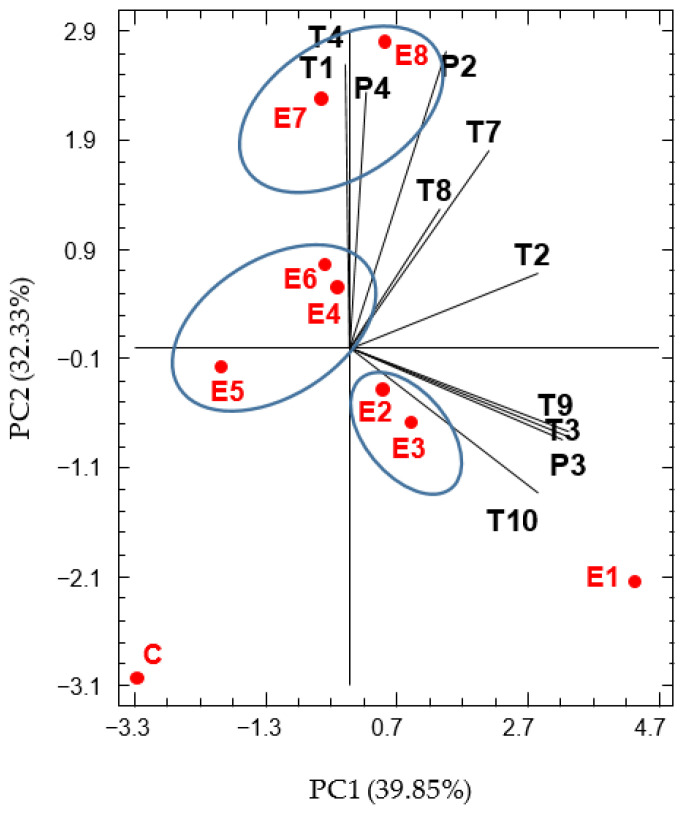
Principal component analysis of VOCs of wines. VOC codes: T1: α-Terpineol; T2: Eucalyptol; T3: Terpinen-4-ol; T4: Citronellal; T7: α-Pinene; T8: Citronellol; T9: Linalool; T10: Limonene; P2: Benzyl alcohol; P3: Eugenol; P4: Methyl eugenol. Wine codes: E1–E8: enzyme-treated wines; C: control wine. For this analysis, VOCs with statistically significant differences (*p* < 0.05) among the wines have been used.

To show the relationship between the released VOCs and the commercial enzyme preparation, a Principal Component Analysis (PCA) was performed using VOCs where significant values were detected. Analyzing the percentages of variance, the first two principal components (PC) accounted for 72.18% of the total variance, describing the main variation in the variables between the different types of enzyme-treated wines (Figure 2). 

The PC1 was positively correlated with almost all the VOCs, except for α-terpineol and citronellal, which were slightly in the negative values. The distribution of the aromatic compounds in positive and negative values of PC2 separates them into two differentiated groups. The positive values of PC2 correlated with positive loadings of some terpenes (α-terpineol, citronellal, methyl-eugenol, α-pinene, citronellol, and eucalyptol) and benzyl alcohol, while negative values correlated with an increment in terpinen-4-ol, linalool, limonene, and eugenol.

The samples treated with the enzyme preparations E7 and E8 were in the positive values of PC2 very close to each other. They can be grouped together because they both present similar aromatic profiles characterized by high levels of methyl eugenol, α-terpineol, benzyl alcohol, and citronellal. These compounds may confer floral and fruity notes to the wines. The sample accounting for the highest concentrations of terpinen-4-ol, linalool, limonene, and eugenol corresponds to treatment with the commercial preparation E1. Despite the fact that the treated samples with E2 and E3 were also plotted in this quadrant, they present lower levels in these compounds than E1 and a similar aromatic profile between them. On the one hand, these compounds confer floral and fruity notes in the wine. On the other hand, they contribute to balsamic, clove, and wood aromas characteristic of terpinen-4-ol and eugenol [33,34]. Samples E4, E5, and E6, located at negative values of PC1, can be grouped by their similar aromatic profile. Significant differences can be found in the aromatic profiles compared to the control. However, they do not stand out for a differentiated aromatic profile, showing similar levels in most of the compounds to E2 and E3, except for a significant decrease in limonene and eugenol (Table 3). The control sample was scattered to the negative values of PC1 and PC2 and presented the lowest levels of the terpenes and benzyl alcohol. 

These results demonstrate the possibility of enhancing the floral, fruity, and/or balsamic characters of the wines by choosing the appropriate commercial glycosidase preparations. Further investigations are required to establish correlations between the selective enhancement of the wine aroma and the composition of glycosidases in commercial enzyme preparations. It is also necessary to conduct a descriptive sensory analysis and a consumer analysis to corroborate the chromatographic data.

## 3. Materials and Methods

### 3.1. Chemical Reagents and Standards

All reagents were analytical grade, and all solvents were HPLC grade. Anhydrous sodium sulfate was from Panreac Química (Barcelona, Spain). The water was Milli-Q^®^ grade (Millipore, Bedford, MA, USA). The identification and quantification of the volatile compounds by gas chromatography were performed using commercial pure standards from Sigma-Aldrich (St. Louis, MO, USA): α-terpineol, eucalyptol, terpinen-4-ol, citronellal, geraniol, methyl eugenol, nerol, α-pinene, citronellol, linalool, limonene, β-pinene, eugenol, theaspirane, α-ionone, β-ionone, 2-phenylethanol, and benzyl alcohol. As internal standards, we utilized 3-octanol and 4-methyl-2-pentanol (Sigma-Aldrich).

### 3.2. Winemaking Procedure

Verdejo grapes (*Vitis vinifiera* L.) were obtained from the Appellation of Origin Rueda located in the region of Castilla y Leon (Spain). The grapes, harvested manually, were processed in the experimental winery of the Higher Technical School of Agrarian Engineering of Palencia at the University of Valladolid. Verdejo grapes were destemmed and crushed, and the must (density of 1.090 g/mL, total acidity of 4.8 g/L expressed as tartaric acid, pH 3.37, and probable alcoholic degree of 12.2% *v*/*v*) was fermented in two stainless-steel tanks (200 L). The concentration of sulfur dioxide in the must was adjusted to 20 mg/L by the addition of potassium metabisulfite, while the total acidity was adjusted to 5.4 g/L using tartaric acid. The must was inoculated with *S. cerevisiae* (Viacell^®^ BAY, Lallemand, Montreal, QC, Canada, 25 g/hL). The density and the temperature of the must wine were monitored during fermentation at 16–18 °C for approximately three weeks until the sugar content decreased to less than 5 g/L. The wine was then clarified by settling at 4 °C for one week. The two tanks were then combined and divided into nine lots of ten liters each. Eight lots were added with eight different enzyme preparations at the maximum concentrations recommended by the manufacturer, while the last lot was used as a control (C) (Table 4). After an enzyme treatment of 15 days at 20 °C, the enzyme reaction was stopped by adding bentonite (70 g/hL, Superbenton^TM^ DC, Dal Cin Gildo Spa, Milan, Italy) as a clarificant. The wine clarification process lasted for one week at 4 °C, followed by the wines being racked, stored at 4 °C, and analyzed. This procedure was performed in triplicate.

### 3.3. Assay of β-Glucosidase Activity under Different Enological Conditions

To evaluate β-glucosidase efficiency under white winemaking conditions (free sulfur dioxide, reducing sugars, and alcoholic degree), a Box–Behnken design with 15 runs was performed (Table 1). The modification of the base wine (runs 1–15) (total acidity: 5.7 g/L of tartaric acid; volatile acidity: 0.29 g/L acetic acid; free sulfur dioxide: 12 mg/L; total sulfur dioxide: 55 mg/L, pH: 3.56; alcoholic degree: 10.0% (*v*/*v*), reducing sugars: 1.8 g/L glucose) was achieved by adding different concentrations of potassium bitartrate, glucose, and ethanol.

β-glucosidase activity was determined by incubating a reaction mixture containing 3 mL of Verdejo wine (with different concentrations of free sulfur dioxide, reducing sugars, and alcoholic degree), 1 mL of 0.01 M 4-nitrophenyl-β-d-glucopyranoside (pNPG), and 1 mL of the enzyme solution at the concentration specified in the previous section. The enzyme reaction was stopped after 1 h of incubation at 37 °C by adding 8 mL of 0.1 M THAM (Tris-hydroxymethylaminomethane) at a pH of 12.0. The solution was filtered and measured spectrophotometrically at 410 nm to determine the absorbance of the resulting color caused by p-nitrophenol (pNP). Controls, in which the substrate was added after the addition of the buffer at pH 12.0, were included to show any non-enzyme activity [39]. One enzyme unit (U) was defined as the amount of enzyme necessary for the hydrolysis of 1 µmol/min of substrate under the settled experimental conditions. 

### 3.4. Analytical Procedure of Volatile Organic Compounds

Terpenes, C13-norisoprenoids, and phenols present in the wine samples were fractioned by selective retention on an AccuBond II ODS-C18 cartridge (500 mg, 6 mL, Agilent Technologies, Inc., Santa Clara, CA, USA) as previously described [40]. A solid-phase extraction vacuum device (Thermo Fisher Scientific, Inc., Waltham, MA, USA) was used to carry out the extractions. Fifteen milliliters of Milli-Q^®^ water and 1 mL of internal standard (3-octanol, 10 mg/mL) were added to 15 mL of wine. This mixture was eluted through the cartridge ODS-C18 (previously activated with 3 mL of methanol and 5 mL of Milli-Q^®^ water). After elution, the cartridge was washed with 12 mL of Milli-Q^®^ water to eliminate sugars, acids, and other water-soluble compounds, and the bound compounds were eluted with 10 mL of dichloromethane-pentane (1:2, *v*/*v*). This fraction, after drying under anhydrous sodium sulfate, was evaporated at 25 °C (until 0.5 mL). After concentration, the sample was ready for Gas Chromatography-Flame Ionization Detector (GC-FID) analysis. The gas chromatograph was an HP 6890 (Agilent Technologies, Inc.) equipped with an automatic injector and an FID detector. The capillary column was an HP-INNOWax (30 m × 0.25 mm internal diameter and 0.25 μm film thickness, Agilent Technologies, Inc.). Chromatographic conditions were as follows: injector temperature, 250 °C; detector temperature, 260 °C; N_2_ at 1.2 mL/min as carrier gas; hydrogen at 40 mL/min and air at 360 mL/min as detector gas; oven temperature program, 80 °C for 5 min, and raised at 3.5 °C/min up to 150 °C, and then at 20 °C/min up to 190 °C; injection (1 µL) in splitless mode (60 s).

The identification and quantification of individual volatiles were performed using pure commercial standards. Identification of the peaks was achieved by comparison of the GC retention times with those of the standards. The relative response areas for each of the volatile compounds to the internal standard were calculated and interpolated into the corresponding calibration graphs. For the calibration, the dissolution of the standards was prepared in 13% ethanol (*v*/*v*) with 5 g/L tartaric acid and the corresponding internal standard in the same concentration as in the samples. Calibration curves were drawn for each standard at eight different concentration levels. The measurements of all samples and standards were performed in triplicate.

### 3.5. Statistical Analysis

The Box–Behnken experimental design, Analysis of Variance (ANOVA), and Principal Component Analysis (PCA) were performed using the computer program Statgraphics Centurion version 19 (Statgraphics Technologies, Inc., The Plains, VA, USA).

## 4. Conclusions

The wide range of enzymes present in the commercial preparations available for oenological application requires winemakers to be aware of their impact on the volatile composition of the wine when selecting the most appropriate preparation. Eight commercial enzyme preparations were assessed on Verdejo grapes to evaluate their potential to enhance the aromatic profile of the wine. All the preparations demonstrated resistance under the tested winemaking conditions, as their efficacy remained unaltered by the presence of reducing sugars, alcoholic degree, or sulfur dioxide. The chemical composition analysis of the treated wines indicated an elevation in the reducing sugars, along with decreased levels of free sulfur dioxide as compared to the control. No effect on total sulfur dioxide, pH, or total and volatile acidity was detected. Interestingly, all the treated wines showed increased levels of terpenes and benzyl alcohol, providing wines with floral, fruity, and/or balsamic notes. The efficiency of the commercial preparations varied, as differences in their ability to release individual compounds were found. According to our data, the wines treated with E1 have a clearly differentiated aromatic profile, as do the wines treated with the enzyme preparations E7 and E8, which can be grouped together because of the similarity of their aromatic profiles. On the one hand, the wines obtained after treatment with the commercial preparation E1 accounted for the highest concentrations of terpinen-4-ol, linalool, limonene, and eugenol. These compounds are responsible for the floral and fruity notes in the wine and for the balsamic, clove, and woody aromas characteristic of terpinen-4-ol and eugenol. On the other hand, the wines treated with the enzyme preparations E7 and E8 are characterized mainly by compounds that can give floral and fruity notes to the wines. Although the activity of β-glucosidase varied in each preparation, no correlation was found with the final aromatic profile detected. Therefore, the results indicate that, depending on the desired characteristics of the final wine, winemakers have the possibility to use a concrete commercial enzyme preparation to achieve this goal. Further studies are required for a better understanding of the effect of specific enzymes on the overall flavor of the wines.

## Figures and Tables

**Table 1:** β-glucosidase activity of the commercial preparations tested under different winemaking conditions.

Box-Behnken Design				β-Glucosidase Activity (U)							
Trial Nº	FS	RS	AG	E1	E2	E3	E4	E5	E6	E7	E8
1	12	1.8	12.5	33.231	29.296	3.810	68.004	15.411	38.813	29.598	26.562
2	60	1.8	12.5	33.288	28.178	3.845	70.898	13.565	37.606	26.313	27.414
3	12	10.0	12.5	33.469	30.362	4.030	68.199	13.565	39.169	26.935	28.267
4	60	10.0	12.5	33.483	31.001	3.636	72.993	12.109	39.399	26.473	29.687
5	12	5.9	10.0	33.409	30.770	4.076	68.359	13.458	40.181	25.816	27.397
6	60	5.9	10.0	33.554	29.847	4.186	69.584	14.825	38.352	25.088	25.994
7	12	5.9	15.0	33.551	31.711	4.296	71.289	14.293	39.488	26.509	25.870
8	12	5.9	15.0	33.522	31.001	3.987	68.359	13.370	40.092	27.982	27.112
9	60	1.8	10.0	33.320	30.539	3.952	70.720	13.654	36.381	25.337	25.230
10	36	10.0	10.0	33.398	31.480	4.037	68.696	12.180	38.955	25.337	24.240
11	36	1.8	15.0	33.373	31.818	3.945	68.199	12.624	37.464	26.935	25.337
12	36	10.0	15.0	33.377	32.972	4.169	72.567	14.275	38.121	30.291	29.616
13	36	5.9	12.5	33.153	30.308	4.364	65.345	15.500	38.227	33.771	28.196
14	36	5.9	12.5	33.242	28.125	4.520	66.299	16.335	39.950	32.759	30.415
15	36	5.9	12.5	33.323	27.858	4.538	67.791	15.749	41.779	33.043	29.687
				**33.380 ± 0.122 ^e^**	**30.351 ± 1.475 ^d^**	**4.093 ± 0.256 ^a^**	**69.153 ± 2.167 ^g^**	**14.061 ± 1.296 ^b^**	**38.932 ± 1.324 ^f^**	**28.146 ± 3.000 ^c^**	**27.402 ± 1.888 ^c^**

FS: free sulfur dioxide (mg/L); RS: reducing sugars (g/L); AG: alcoholic grade (% *v*/*v*). Average ± standard deviation in bold. Different letters mean statistically significant differences at *p* < 0.05 between samples.

**Table 2:** General composition of the enzyme-treated and control wines.

Wine	FS	TS	pH	TA	VA	RS
E1	34 ± 3 ^a^	110 ± 10	3.40 ± 0.21	5.4 ± 0.1	0.26 ± 0.03	2.9 ± 0.1 ^c^
E2	32 ± 1 ^a^	108 ± 9	3.32 ± 0.19	5.3 ± 0.2	0.26 ± 0.02	3.2 ± 0.2 ^c^
E3	36 ± 3 ^a^	123 ± 5	3.35 ± 0.16	5.3 ± 0.1	0.28 ± 0.02	3.0 ± 0.2 ^c^
E4	31 ± 1 ^a^	124 ± 6	3.38 ± 0.22	5.4 ± 0.2	0.27 ± 0.01	3.0 ± 0.2 ^c^
E5	30 ± 2 ^a^	129 ± 7	3.36 ± 0.20	5.3 ± 0.1	0.27 ± 0.02	3.1 ± 0.2 ^c^
E6	31 ± 1 ^a^	132 ± 8	3.30 ± 0.12	5.5 ± 0.2	0.25 ± 0.02	2.8 ± 0.1 ^c^
E7	30 ± 2 ^a^	118 ± 7	3.33 ± 0.11	5.4 ± 0.1	0.26 ± 0.02	2.8 ± 0.1 ^c^
E8	29 ± 1 ^a^	116 ± 11	3.29 ± 0.18	5.4 ± 0.1	0.25 ± 0.02	2.7 ± 0.0 ^b^
C	40 ± 2 ^b^	126 ± 9	3.25 ± 0.20	5.4 ± 0.1	0.26 ± 0.03	1.3 ± 0.2 ^a^

FS: free sulfur dioxide (mg/L); TS: total sulfur dioxide (mg/L); TA: total acidity (g/L tartaric acid); VA: volatile acidity (g/L acetic acid); RS: reducing sugars (g/L). Different letters mean statistically significant differences at *p* < 0.05 among the samples. Data expressed as mean value ± standard deviation.

**Table 3:** Volatile organic compounds (VOCs).

Code	Compounds	E1	E2	E3	E4	E5	E6	E7	E8	C
Terpenes										
T1	α-Terpineol	6.14 ± 1.12 ^b^	17.31 ± 0.43 ^e^	11.05 ± 0.91 ^c^	15.88 ± 0.82 ^de^	15.46 ± 0.53 ^de^	10.70 ± 0.25 ^c^	15.41 ± 0.87 ^de^	14.83 ± 1.11 ^d^	2.47 ± 0.17 ^a^
T2	Eucalyptol	9.17 ± 0.72 ^c^	6.76 ± 0.14 ^ab^	7.13 ± 0.86 ^b^	5.89 ± 0.69 ^ab^	6.57 ± 0.79 ^ab^	6.85 ± 0.62 ^ab^	6.93 ± 0.85 ^ab^	8.94 ± 0.18 ^c^	5.64 ± 0.11 ^a^
T3	Terpinen-4-ol	29.94 ± 8.82 ^c^	14.78 ± 1.59 ^b^	13.81 ± 0.62 ^b^	14.16 ± 2.03 ^b^	12.75 ± 1.68 ^b^	9.13 ± 0.35 ^b^	8.51 ± 0.97 ^b^	13.19 ± 3.17 ^b^	5.84 ± 0.36 ^a^
T4	Citronellal	0.03 ± 0.02 ^a^	0.04 ± 0.02 ^a^	0.07 ± 0.04 ^a^	0.08 ± 0.00 ^a^	0.07 ± 0.00 ^a^	0.42 ± 0.02 ^b^	0.42 ± 0.00 ^b^	0.44 ± 0.02 ^b^	nd
T5	Geraniol	0.03 ± 0.02	0.09 ± 0.06	0.08 ± 0.01	0.08 ± 0.06	0.08 ± 0.06	0.05 ± 0.04	0.08 ± 0.06	0.07 ± 0.05	0.02 ± 0.00
T6	Nerol	11.91 ± 7.73	8.10 ± 2.83	7.85 ± 2.60	7.94 ± 2.74	6.38 ± 2.21	6.6 ± 1.72	6.44 ± 1.63	7.68 ± 2.69	6.52 ± 0.18
T7	α-Pinene	23.60 ± 0.24 ^b^	23.77 ± 0.2 ^b^	23.73 ± 0.15 ^b^	23.62 ± 0.08 ^b^	23.62 ± 0.05 ^b^	23.74 ± 0.23 ^b^	23.53 ± 0.26 ^b^	23.46 ± 0.12 ^b^	12.81 ± 0.35 ^a^
T8	Citronellol	9.55 ± 2.19 ^abc^	8.32 ± 0.96 ^ab^	13.19 ± 0.59 ^bc^	15.81 ± 1.07 ^c^	nd	12.44 ± 2.73 ^abc^	10.41 ± 4.65 ^abc^	14.85 ± 1.18 ^c^	6.99 ± 0.91 ^a^
T9	Linalool	22.63 ± 1.17 ^d^	10.98 ± 6.50 ^bc^	12.09 ± 2.10 ^bc^	12.88 ± 3.20 ^c^	4.28 ± 0.59 ^a^	8.08 ± 2.58 ^abc^	8.11 ± 0.26 ^abc^	10.98 ± 0.52 ^bc^	6.76 ± 2.36 ^ab^
T10	Limonene	0.06 ± 0.00 ^c^	0.04 ± 0.01 ^b^	0.04 ± 0.00 ^b^	0.02 ± 0.01 ^a^	nd	0.02 ± 0.01 ^a^	0.02 ± 0.01 ^a^	nd	nd
T11	β-Pinene	45.24 ± 0.23	19.47 ± 10.53	22.27 ± 0.21	25.97 ± 0.27	14.67 ± 1.80	13.52 ± 0.94	21.19 ± 15.09	18.85 ± 0.79	14.23 ± 1.86
C13-Norisoprenoids										
N1	Theaspirane	0.06 ± 0.02	0.04 ± 0.00	0.05 ± 0.01	0.05 ± 0.01	0.04 ± 0.01	0.05 ± 0.01	0.06 ± 0.02	0.05 ± 0.01	0.03 ± 0.00
N2	α-Ionone	0.17 ± 0.02	0.18 ± 0.08	0.08 ± 0.02	0.10 ± 0.00	0.10 ± 0.02	0.08 ± 0.02	0.09 ± 0.02	0.08 ± 0.01	0.08 ± 0.01
N3	β-Ionone	0.04 ± 0.01	0.02 ± 0.00	0.02 ± 0.01	0.03 ± 0.01	0.03 ± 0.00	0.02 ± 0.01	0.02 ± 0.00	0.02 ± 0.00	0.02 ± 0.01
Phenols and derivates										
P1	2-Phenylethanol	0.05 ± 0.00	0.03 ± 0.00	0.03 ± 0.01	0.04 ± 0.01	0.03 ± 0.00	0.03 ± 0.00	0.04 ± 0.01	0.05 ± 0.01	nd
P2	Benzyl alcohol	17.63 ± 0.64 ^b^	20.79 ± 1.59 ^bc^	20.53 ± 1.68 ^bc^	20.88 ± 2.17 ^bc^	18.39 ± 5.31 ^b^	21.74 ± 1.34 ^bc^	28.20 ± 2.68 ^c^	26.61 ± 3.95 ^c^	2.49 ± 0.39 ^a^
P3	Eugenol	42.52 ± 14.62 ^c^	23.4 ± 4.12 ^b^	26.39 ± 2.65 ^b^	13.15 ± 3.53 ^a^	10.28 ± 1.87 ^a^	21.52 ± 1.50 ^b^	16.9 ± 3.02 ^b^	18.15 ± 0.37 ^b^	11.03 ± 0.37 ^a^
P4	Methyl eugenol	9.52 ± 1.08 ^a^	6.97 ± 0.58 ^a^	6.00 ± 1.73 ^a^	12.55 ± 6.61 ^a^	6.86 ± 1.75 ^a^	8.22 ± 2.88 ^a^	19.63 ± 0.11 ^b^	22.53 ± 1.86 ^b^	8.25 ± 0.83 ^a^

Data expressed in μg/L (mean value ± standard deviation). In the same row, different letters indicate statistically significant differences among samples (*p* < 0.05). nd: not detected.

**Table 4:** List of assayed commercial preparations with β-glucosidase activity.

Code	Commercial Name	Declared Enzyme Activities	Dose
E1	Scottzyme^®^ βG (Scott Laboratories, Petaluma, CA, USA)	Pectinases and β-glucosidase	5 g/hL
E2	Lallzyme Beta^TM^ (Lallemand, Montreal, QC, Canada)	Polygalacturonase and β-glucosidase	5 g/hL
E3	Endozyme^®^ β split (AEB Group, Barcelona, Spain)	Cellulases, pectinases, and β-glucosidase	5 g/hL
E4	Rapidase^®^ Revelation Aroma (AR2000) (DSM Food, AX Delft, The Netherlands)	Pectinase and β-glucosidase	3 g/hL
E5	Depectil AR (Martin Vialatte, Magenta, France)	Pectinase and β-glucosidase	10 g/hL
E6	Novarom^®^ Blanc (Novozymes, Bagsværd, Denmark)	Polygalacturonase and β-glucosidase	10 g/hL
E7	Enovin Varietal (Agrovin, Alcázar de San Juan, Spain)	Pectinases and glycosidases	10 g/hL
E8	Lafazym^®^ Arom (Laffort, Bordeaux, France)	Pectinase and β-glucosidase	10 g/hL

## Data Availability

Data are contained within the article.

## References

[1] Pogorzelski E., Wilkowska A. (2007). Flavour Enhancement through the Enzymatic Hydrolysis of Glycosidic Aroma Precursors in Juices and Wine Beverages: A Review. Flavour Fragance J..

[2] Cabaroglu T., Selli S., Canbas A., Leproutre J.P., Günata Z. (2003). Wine Flavor Enhancement through the Use of Exogenous Fungal Glycosidases. Enzym. Microb. Technol..

[3] Mateo J.J., Jiménez M. (2000). Monoterpenes in Grape Juice and Wines. J. Chromatogr. A.

[4] Liu J.B., Zhu X.L., Ullah N., Tao Y.S. (2017). Aroma Glycosides in Grapes and Wine. J. Food Sci..

[5] Maicas S., Mateo J.J. (2005). Hydrolysis of Terpenyl Glycosides in Grape Juice and Other Fruit Juices: A Review. Appl. Microbiol. Biotechnol..

[6] Wilkowska A., Pogorzelski E. (2017). Aroma Enhancement of Cherry Juice and Wine Using Exogenous Glycosidases from Mould, Yeast and Lactic Acid Bacteria. Food Chem..

[7] Ugliano M., Bartowsky E.J., McCarthy J., Moio L., Henschke P.A. (2006). Hydrolysis and Transformation of Grape Glycosidically Bound Volatile Compounds during Fermentation with Three *Saccharomyces* Yeast Strains. J. Agric. Food Chem..

[8] Liang Z., Fang Z., Pai A., Luo J., Gan R., Gao Y., Lu J., Zhang P. (2022). Glycosidically Bound Aroma Precursors in Fruits: A Comprehensive Review. Crit. Rev. Food Sci. Nutr..

[9] de Morais Souto B., Florentino Barbosa M., Marinsek Sales R., Conessa Moura S., de Rezende Bastos Araújo A., Ferraz Quirino B. (2023). The Potential of β-Glucosidases for Aroma and Flavor Improvement in the Food Industry. Microbe.

[10] Wang Y., Zhang C., Li J., Xu Y. (2013). Different Influences of β-Glucosidases on Volatile Compounds and Anthocyanins of Cabernet Gernischt and Possible Reason. Food Chem..

[11] Zhang P., Zhang R., Sirisena S., Gan R., Fang Z. (2021). Beta-Glucosidase Activity of Wine Yeasts and Its Impacts on Wine Volatiles and Phenolics: A Mini-Review. Food Microbiol..

[12] Gil J.V., Manzanares P., Genovés S., Vallés S., González-Candelas L. (2005). Over-Production of the Major Exoglucanase of *Saccharomyces cerevisiae* Leads to an Increase in the Aroma of Wine. Int. J. Food Microbiol..

[13] Baffi M.A., Tobal T., Ghilardi Lago J.H., Boscolo M., Gomes E., Da-Silva R. (2013). Wine Aroma Improvement Using a β-Glucosidase Preparation from *Aureobasidium pullulans*. Appl. Biochem. Biotechnol..

[14] Baffi M.A., Tobal T., Lago J.H.G., Leite R.S.R., Boscolo M., Gomes E., Da-Silva R. (2011). A Novel β-Glucosidase from *Sporidiobolus pararoseus*: Characterization and Application in Winemaking. J. Food Sci..

[15] Zhu F.-M., Du B., Li J. (2014). Aroma Enhancement and Enzymolysis Regulation of Grape Wine Using β-Glycosidase. Food Sci. Nutr..

[16] Gao P., Sam F.E., Zhang B., Peng S., Li M., Wang J. (2022). Enzymatic Characterization of Purified β-Glucosidase from Non-*Saccharomyces* Yeasts and Application on Chardonnay Aging. Foods.

[17] Zhu W., Zhang W., Qin T., Liao J., Zhang X. (2022). Effects of Purified β-Glucosidases from *Issatchenkia terricola*, *Pichia kudriavzevii*, *Metschnikowia pulcherrima* on the Flavor Complexity and Typicality of Wines. J. Fungi.

[18] Fia G., Olivier V., Cavaglioni A., Canuti V., Zanoni B. (2016). Side Activities of Commercial Enzyme Preparations and Their Influence on the Hydroxycinnamic Acids, Volatile Compounds and Nitrogenous Components of White Wine. Aust. J. Grape Wine Res..

[19] Sánchez-Palomo E., Diaz-Maroto M., González-Viñas M., Pérez-Coello M. (2005). Aroma Enhancement in Wines from Different Grape Varieties Using Exogenous Glycosidases. Food Chem..

[20] Palmeri R., Spagna G. (2007). β-Glucosidase in Cellular and Acellular Form for Winemaking Application. Enzym. Microb. Technol..

[21] Bezus B., de Ovalle S., González-Pombo P., Cavalitto S., Cavello I. (2023). Production and Characterization of a Novel Cold-Active β-Glucosidase and Its Influence on Aromatic Precursors of Muscat Wine. Food Biosci..

[22] da Silva R.R., da Conceição P.J.P., de Menezes C.L.A., de Oliveira Nascimento C.E., Machado Bertelli M., Pessoa Júnior A., de Souza G.M., da Silva R., Gomes E. (2019). Biochemical Characteristics and Potential Application of a Novel Ethanol and Glucose-Tolerant β-Glucosidase Secreted by *Pichia guilliermondii* G1.2. J. Biotechnol..

[23] Esbensen K.H., Guyot D., Westad F., Houmoller L.P. (2002). Multivariate Data Analysis: In Practice: An Introduction to Multivariate Data Analysis and Experimental Design.

[24] Bosque-Sendra J.M., Pescarolo S., Cuadros-Rodríguez L., García-Campaña A.M., Almansa-López E.M. (2001). Optimizing Analytical Methods Using Sequential Response Surface Methodology: Application to the Pararosaniline Determination of Formaldehyde. Fresenius J. Anal. Chem..

[25] Ketudat Cairns J., Esen A. (2010). β-Glucosidases. Cell Mol. Life Sci..

[26] Mangas-Sánchez J., Adlercreutz P. (2015). Enzymatic Preparation of Oligosaccharides by Transglycosylation: A Comparative Study of Glucosidases. J. Mol. Catal. B Enzym..

[27] Barbagallo R.N., Spagna G., Palmeri R., Restuccia C., Giudici P. (2004). Selection, Characterization and Comparison of β-Glucosidase from Mould and Yeasts Employable for Enological Applications. Enzym. Microb. Technol..

[28] Scutarașu E.C., Luchian C.E., Vlase L., Nagy K., Colibaba L.C., Trinca L.C., Cotea V.V. (2022). Influence Evaluation of Enzyme Treatments on Aroma Profile of White Wines. Agronomy.

[29] Martino A., Schiraldi C., Di Lazzaro A., Fiume I., Spagna G., Pifferi P.G., De Rosa M. (2000). Improvement of the Flavour of Falanghina White Wine Using a Purified Glycosidase Preparation from *Aspergillus niger*. Process Biochem..

[30] Guerrero R.F., Cantos-Villar E. (2015). Demonstrating the Efficiency of Sulphur Dioxide Replacements in Wine: A Parameter Review. Trends Food Sci. Technol..

[31] OIV (2021). SO_2_ and Wine: A Review.

[32] Parker M., Capone D.L., Francis I.L., Herderich M.J. (2018). Aroma Precursors in Grapes and Wine: Flavor Release during Wine Production and Consumption. J. Agric. Food Chem..

[33] Perestrelo R., Silva C., Camara J.S. (2019). Madeira Wine Volatile Profile. A Platform to Establish Madeira Wine Aroma Descriptors. Molecules.

[34] Tao Y.-S., Li H. (2009). Active Volatiles of Cabernet Sauvignon Wine from Changli County. Health.

[35] Fang Y., Qian M. (2005). Aroma Compounds in Oregon Pinot Noir Wine Determined by Aroma Extract Dilution Analysis (AEDA). Flavour Fragance J..

[36] Yuan F., Cheng K., Gao J., Pan S. (2018). Characterization of Cultivar Differences of Blueberry Wines Using GC-QTOF-MS and Metabolic Profiling Methods. Molecules.

[37] Tan K.H., Nishida R. (2012). Methyl Eugenol: Its Occurrence, Distribution, and Role in Nature, Especially in Relation to Insect Behavior and Pollination. J. Insect Sci..

[38] González-Barreiro C., Rial-Otero R., Cancho-Grande B., Simal-Gándara J. (2015). Wine Aroma Compounds in Grapes: A Critical Review. Crit. Rev. Food Sci. Nutr..

[39] Dong R., Abdelkerim-Ouba D., Liu D., Ma X., Wang S. (2023). Impacts of Partial Substitution of Chemical Fertilizer with Organic Manure on the Kinetic and Thermodynamic Characteristics of Soil β-Glucosidase. Agronomy.

[40] Diéguez S.C., de la Peña M.L.G., Gómez E.F. (2003). Approaches to Spirit Aroma: Contribution of Some Aromatic Compounds to the Primary Aroma in Samples of Orujo Spirits. J. Agric. Food Chem..

